# *Diplorickettsia* Bacteria in an *Ixodes scapularis* Tick, Vermont, USA

**DOI:** 10.3201/eid2605.191135

**Published:** 2020-05

**Authors:** Carter Merenstein, Jeremy Ward, David Allen

**Affiliations:** Boston University School of Medicine, Boston, Massachusetts, USA (C. Merenstein);; Middlebury College, Middlebury, Vermont, USA (J. Ward, D. Allen)

**Keywords:** *Ixodes scapularis*, *Diplorickettsia*, tick, microbiome, bacteria, blacklegged tick, 16S rRNA, Vermont, United States, vector-borne infections

## Abstract

An unexpected *Diplorickettsia* species closely related to the tickborne pathogen *D. massieliensis* was found in the microbiome of an *Ixodes scapularis* tick in Vermont, USA. This evidence of *Diplorickettsia* in North American ticks suggests a need for disease surveillance using molecular screening of ticks and serologic studies of humans.

The blacklegged tick, *Ixodes scapularis*, is a generalist arthropod ectoparasite that serves as a vector for an array of common human pathogens; novel disease-causing microbes have been discovered consistently in the tick for the past several decades. Tickborne bacterial infections causing illnesses such as anaplasmosis, *Borrelia miyamotoi* disease, and ehrlichiosis have all emerged in the United States in recent years (*1**–**3*), and it has been estimated that as many as half of all tickborne illnesses are caused by unknown pathogens (*4*).

We used 16S rRNA sequencing to survey for bacterial pathogens in *I. scapularis* ticks in western Vermont, USA. We collected ticks by drag sampling along 100 m transects using a 1 m^2^ square of white denim. We collected ticks from 6 deciduous forest sites in Addison and Chittenden counties, Vermont (Appendix Table 1), during May–July 2015. We extracted DNA from ticks using a phenol-chloroform extraction (*5*). In total, we extracted DNA from 97 ticks, 20 of which we selected based on DNA quality and quantity for 16S rDNA sequencing at Hudson Alpha Genomic Services Laboratory (Huntsville, AL, USA). We PCR amplified the V3 and V4 regions of the 16S rRNA gene from these ticks and 1 blank using 341F and 875R primers (*6*) and sequenced them on a MiSeq platform (Illumina, https://www.illumina.com), yielding a total of 15,302,568 reads. We used the DADA2 R package to identify amplicon sequence variants (ASVs) and assign taxonomy (*7*) (Appendix Figure 1). We used default settings in the DADA2 pipeline; however, we estimated error rates using the first 10 billion base pairs. 

In a single adult male tick, an ASV assigned to the genus *Diplorickettsia* comprised 82% of the microbiome sequencing reads. The genus *Diplorickettsia* was originally defined by the species *D. massiliensis*, discovered in *I. ricinus* ticks in Europe (*8*). The ASV we identified shared 425 of 427 nt in the sequenced V3–V4 region of the 16S rRNA gene with the reference sequence of *D. massiliensis* strain 20B (GenBank accession no. NR_117407.1) and was more closely related to this strain than any other previously sequenced *Diplorickettsia* in the National Center for Biotechnology Information (NCBI) nucleotide database (Figure). This tick was collected at the Sunny Hollow Colchester site (coordinates 44.518353°, −73.17112°) (Appendix Table 1).

**Figure:**
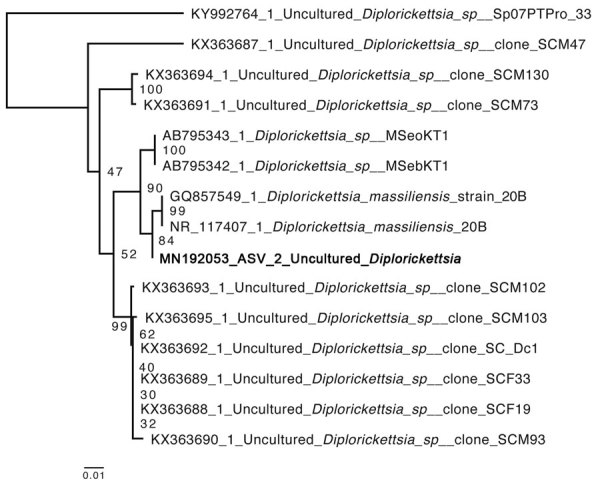
Neighbor-joining phylogenetic tree of a MAFFT alignment (https://mafft.cbrc.jp/alignment/server) of the V3–V4 region of the *Diplorickettsia* 16S rRNA gene, including the novel amplicon sequence variant identified in Vermont, USA (bold). A total of 427 bases were aligned and 363 conserved sites were used for neighbor-joining phylogeny, with 100 bootstrap iterations. The 341F and 875R primers were used to amplify these regions (*6*). Default alignment parameters were used for alignment and generation of phylogenetic tree. Numbers at nodes indicate bootstrap values after 1,000 bootstrapping iterations. GenBank accession numbers are indicated. Scale bar represents average number of substitutions per site.

*D. massiliensis* has been identified as a possible human pathogen; of patients in a hospital in France, 3 were found seropositive, and 1 found positive by quantitative PCR for the *D. massiliensis rpoB* gene (*9*). Our findings represent evidence of a *Diplorickettsia* bacteria in ticks in North America.

To confirm the presence of *Diplorickittsia* in the positive tick, we designed PCR primers to regions of the *D. massiliensis parC* and *ftsY* genes (Appendix Table 2) using primer-BLAST (https://www.ncbi.nlm.nih.gov/tools/primer-blast). We aligned primers against the NCBI nr database to ensure specific binding to *Diplorickettsia*. We also used DNA from a *Diplorickettsia*-negative tick (as determined by 16S sequencing) as PCR template to serve as a negative control. Successful amplification of regions of both genes confirmed the presence of *Diplorickettsia* DNA in the positive tick (Appendix Figure 2).

We Sanger sequenced amplicons from these PCR tests. We combined forward and reverse reads and trimmed them using the PEAR utility (https://cme.h-its.org/exelixis/web/software/pear). Each sequence showed high identity with previously sequenced *D. massiliensis* reference sequences via gapped alignment (247/252 bp *parC,* 298/310 bp *ftsY*). These results further suggest a close relationship between *Diplorickettsia* species we identified and *D. massiliensis*, but a lack of reference sequences for these genes from other species of *Diplorickettsia* makes it impossible to definitively assign this uncultured specimen to a particular species. 

The high sequence similarity between the *Diplorickettsia* we identified and the previously identified pathogenic variety suggests the need for further study of the pathogenicity of this variant. Many genera of tickborne bacteria contain both pathogenic and nonpathogenic strains, and genetic similarity alone cannot confirm pathogenicity. Future work is needed to isolate this strain of *Diplorickettsia* and determine its ability to infect mammalian hosts and its transmissibility via tick bite. Experiments to test its ability to induce febrile illness in mammals would also help determine if *Diplorickettsia* spp. could cause a clinically significant infection in humans. Furthermore, serologic studies of patients with suspected tickborne diseases in the area surrounding the collection site are necessary to determine if this bacterium has infected persons in Vermont.

In addition, our findings suggest the need for further study of the prevalence of *Diplorickettsia* in North America ticks. We have developed PCR primers (Appendix Table 2) to facilitate future study of this bacterium and have demonstrated via sequencing that these primers accurately amplify their target *Diplorickettsia* genes. We have deposited the partial *Diplorickettsia* 16S rRNA, *ftsY,* and *parC* genes sequenced in this study into the NCBI GenBank database (accession nos. MN192053, MN640996, MN640997). The raw sequencing reads are available through the NCBI sequence read archive (accession no. PRJNA557440).

Appendix Additional information about *Diplorickettsia* bacteria in the microbiome of an *Ixodes scapularis* tick, Vermont, USA.
