# Supplementary material for: Diplorickettsia Bacteria in an Ixodes scapularis Tick, Vermont, USA
Source: Emerg Infect Dis. 2020 May;26(5):1036–8. doi: 10.3201/eid2605.191135 (PMC7181933; doi:10.3201/eid2605.191135)
Supplement: Appendix — Additional information about Diplorickettsia bacteria in the microbiome of an Ixodes scapularis tick, Vermont, USA. [file 19-1135-Techapp-s1.pdf]

Article DOI: https://doi.org/10.3201/eid2605.191135

Diplorickettsia Bacteria in an *Ixodes scapularis* Tick, Vermont, USA

Appendix

Appendix Table 1. Sampling locations for *Ixodes scaularis* tick, Vermont, USA

Site	Latitude, °	Longitude, °	Mean elevation, m
Sunny Hollow Colchester	44.518353	−73.17112	93
Shelbourne Town Forest	44.369066	−73.143851	122
New Haven	44.113699	−73.156602	119
Chipman Hill	44.023385	−73.162146	221
Chipman Hill (Backside)	44.026342	−73.159889	213
Orwell	43.818445	−73.264537	140

Appendix Table 2. Primers used for PCR of novel *Diplorickettsia* bacteria found in *Ixodes scaularis* tick, Vermont, USA

Primer	Melting temperature, °C	Primer sequence	Amplicon length, bt
ftsY forward	60	TCATCGATGGCCAAGCTGTT	386
ftsY reverse	60	TTTCACCTTCGCGAGCTCTT	
parC forward	60	ACTCGACCATCCAAAAGCGT	272
parC reverse	60	TCACCAAACGTGTCGGTTGT	

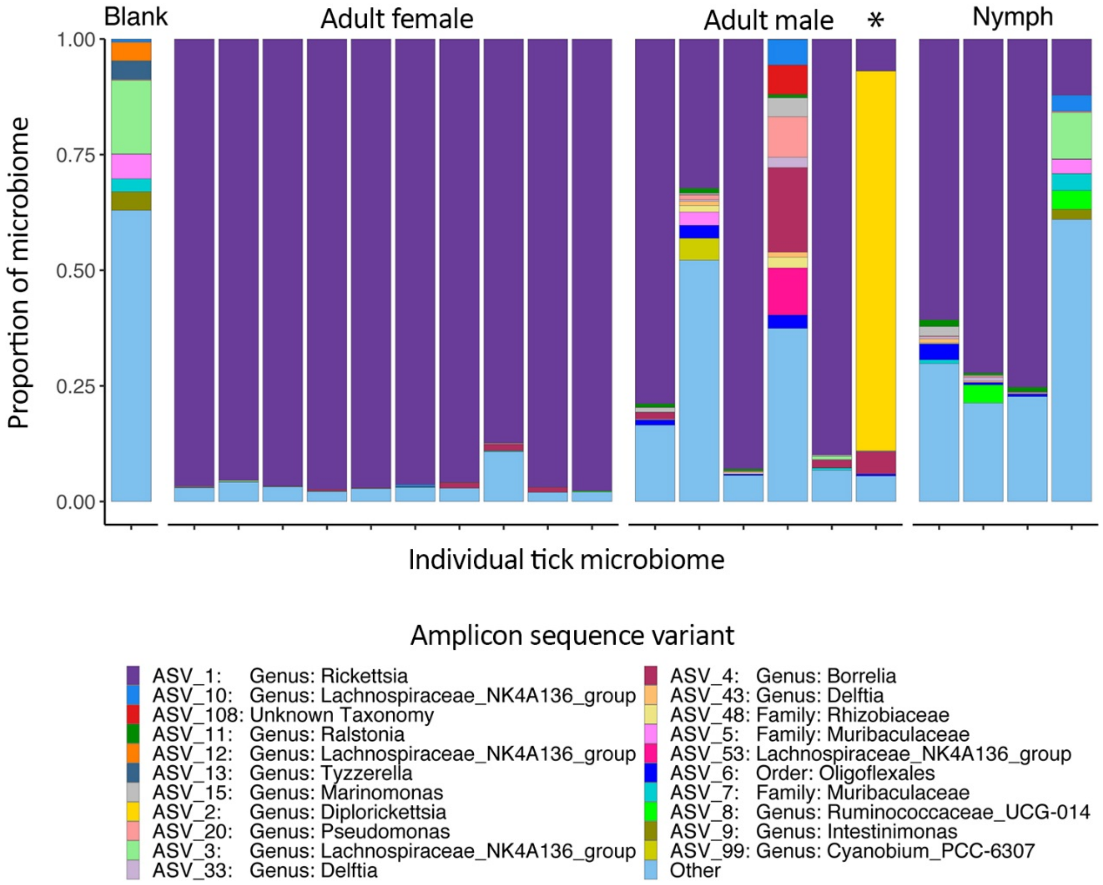

Appendix Figure 1. Microbiome of each tick via 16s RNA sequencing. Each vertical bar represents an individual tick, colored by the proportion of the microbiome made up of each amplicon sequence variant (ASV). For the top 22 ASVs, taxa information is shown as the lowest order to which the ASV can be identified. Asterisk indicates the tick in which *Diplorickettsia* was identified.

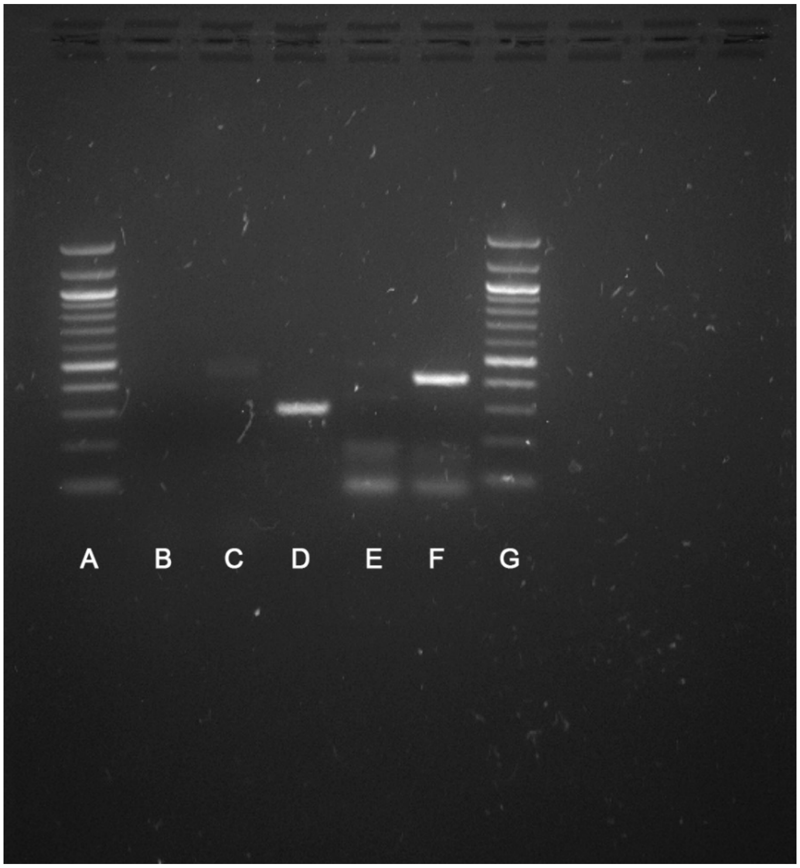

Appendix Figure 2. Presence of *Diplorickettsia* is confirmed by PCR for the *parC* and *ftsY* gene. A) New England Biolabs (https://www.neb.com) 100bp ladder; B) negative control, using the *parC* primers but no DNA; C) *Diplorickettsia*-negative tick with *parC*; D) *Diplorickettsia*-positive tick with *parC* (272 bp amplicon); E) *Diplorickettsia*-negative tick with *ftsY;* F) *Diplorickettsia*-positive tick with *ftsY* (386 bp amplicon); G) NEB 100 bp ladder. We used NEB Phusion 2X master mix for PCR, using 25 μL reactions, 1 μL of tick prep DNA (unquantified); final primer concentration for each sample was 0.4 μM. PCR temperatures were 98°C for 30 sec, followed by 35 cycles of 98°C for 10 sec, 61°C for 15 sec, 72°C for 20 sec, followed by 72°C for 10 min.
